# Comparative effect of angiotensin II type I receptor blockers on serum uric acid in hypertensive patients with type 2 diabetes mellitus: a retrospective observational study

**DOI:** 10.1186/1475-2840-12-159

**Published:** 2013-11-04

**Authors:** Yayoi Nishida, Yasuo Takahashi, Norio Susa, Nobukazu Kanou, Tomohiro Nakayama, Satoshi Asai

**Affiliations:** 1 Division of Genomic Epidemiology and Clinical Trials, Clinical Trials Research Center, Nihon University School of Medicine, 30-1 Oyaguchi-Kami Machi, Itabashi-ku, Tokyo 173-8610, Japan; 2 Department of Biomedical Sciences, Division of Pharmacology, Nihon University School of Medicine, 30-1 Oyaguchi-Kami Machi, Itabashi-ku, Tokyo 173-8610, Japan; 3 Department of Pathology and Microbiology, Division of Laboratory Medicine, Nihon University School of Medicine, 30-1 Oyaguchi-Kami Machi, Itabashi-ku, Tokyo 173-8610, Japan

**Keywords:** ARB monotherapy, Losartan, Valsartan, Telmisartan, Candesartan, Olmesartan, Serum uric acid, Hypertension, Type 2 diabetes mellitus

## Abstract

**Background:**

Angiotensin II type 1 receptor blockers (ARB) are a frequently used class of antihypertensive drug. The ARB losartan is known to decrease the serum uric acid (SUA) level. However, there are very few clinical data comparing the effects of other ARBs on SUA level under the conditions of clinical practice. This study evaluated and compared the long-term effects of monotherapy with five ARBs on SUA level in Japanese hypertensive patients with type 2 diabetes mellitus (DM).

**Methods:**

We identified hypertensive patients with type 2 DM who had been treated with monotherapy with losartan (n = 214), valsartan (n = 266), telmisartan (n = 185), candesartan (n = 458), or olmesartan (n = 192), in whom laboratory data of SUA between November 1, 2004 and July 31, 2011 were available, from the Nihon University School of Medicine’s Clinical Data Warehouse (NUSM’s CDW). We used a propensity-score weighting method and a multivariate regression model to adjust for differences in the background among ARB users, and compared the SUA level. The mean exposure of losartan was 264.7 days, valsartan 245.3 days, telmisartan 235.9 days, candesartan 248.9 days, and olmesartan 234.5 days.

**Results:**

In losartan users, mean SUA level was significantly decreased from baseline, while it was conversely increased in users of other ARBs; valsartan, telmisartan, candesartan, and olmesartan. The mean reduction of SUA level from baseline was significantly greater in losartan users compared with that in other ARB users. Comparison of ARBs other than losartan showed no significant difference in mean change in SUA level from baseline.

**Conclusions:**

Our study showed that losartan had the most beneficial effect on SUA level among five ARBs, and that there was no significant difference in the unfavorable effects on SUA level among four ARBs other than losartan, at least during one year. These findings provide evidence of an effect of ARBs on SUA level, and support the benefit of the use of losartan in hypertensive patients with type 2 DM.

## Introduction

A high concentration of serum uric acid (SUA) is the main cause of gout, and is also associated with the metabolic syndrome, including hypertension and diabetes mellitus (DM) [1-3]. In the report of the US National Health and Nutrition Examination Survey, among patients with gout, 74% had hypertension and 26% had diabetes [4]. Hypertension, DM, and hyperuricemia are mutually related; therefore, regular monitoring of serum uric acid levels is desirable in hypertensive patients with DM [5].

Many patients with hyperuricemia are using antihypertensive agents because hypertension and hyperuricemia are conditions that frequently coexist. The effect of antihypertensive agents on uric acid differs according to their mechanism of action. Beta blockers and thiazide diuretics increase the SUA level whereas alpha-blockers and calcium-channel blockers (CCB) decrease the SUA level [6,7]. The effect of angiotensin II type I receptor blockers (ARBs) on the SUA level differs among drugs. Of ARBs, losartan decreases the SUA level [8-10] via its influence on urate transporter 1 (URAT1) [11-13]. Differing from losartan, valsartan and candesartan have been reported to increase the SUA level in patients with hypertension [14,15].

Several studies have compared the effect of losartan on SUA with that of another drug or placebo. However, these studies were mainly randomized clinical trials (RCTs) in which the efficacy of a treatment in optimal controlled experimental conditions was evaluated, and few studies have compared therapeutic effectiveness using a clinical databases, under the conditions of clinical practice. In addition, few studies have performed a multiple comparison of the effects on SUA level among various ARBs in clinical practice, and few studies have compared the long-term effect of ARB monotherapies on SUA in hypertensive patients with type 2 diabetes mellitus. The aim of this study was to evaluate and compare the long-term effect of five ARB monotherapies; losartan, valsartan, candesartan, telmisartan, and olmesartan, on SUA in Japanese hypertensive patients with type 2 diabetes mellitus, in a real-world setting.

## Materials and methods

### Data source

We used the Nihon University School of Medicine (NUSM) Clinical Data Warehouse (CDW) for this retrospective database study. NUSM’s CDW is a centralized data repository that integrates separate databases, including an order entry database and a laboratory results database, from the hospital information systems at three hospitals affiliated to NUSM. The prescribing data of over 0.5 million patients are linked longitudinally to detailed clinical information such as patient demographics, diagnosis, and laboratory data. Several epidemiological studies examining the effects of drugs on glucose and lipid metabolism and renal function using NUSM’s CDW have been published [16-18].

### Study populations

We identified Japanese type 2 DM patients with mild to moderate hypertension aged over 20 years, who had been newly treated with ARB monotherapy for at least two months between November 1, 2004 and July 31, 2011. DM was diagnosed according to the Committee for the Classification and Diagnosis of Diabetes Mellitus of the Japan Diabetes Society (defined as fasting plasma glucose level ≥126 mg/dl, casual plasma glucose level ≥200 mg/dl, plasma glucose 2 h after 75 g glucose load ≥200 mg/dl, or HbA1c (NGSP) level ≥6.5% [19]).

The five ARBs used in this study were losartan potassium, valsartan, telmisartan, candesartan cilexetil, and olmesartan medoxomil (Table 1). The number of hypertensive type 2 DM patients treated with losartan was 3599, valsartan 6918, telmisartan 4091, candesartan 8730, and olmesartan 5746. We excluded patients who had been treated with other antihypertensive drugs (ARB combination drugs, angiotensin-converting enzyme inhibitor, CCB, diuretic, alpha-blocker, beta-blocker, alpha and beta blocker, alpha-agonist, reserpine, vasodilator, or renin inhibitor) during the study period. We also excluded patients who had been treated with drugs for hyperuricemia or insulin during the study period. The numbers of monotherapy patients in this study were; losartan (n = 214), valsartan (n = 266), telmisartan (n = 185), candesartan (n = 458), and olmesartan (n = 192) (Table 1). The experimental protocol was approved by the Ethical Committee of Nihon University School of Medicine and was conducted in compliance with the ethical guidelines for epidemiological research of the Ministry of Education, Culture, Sports, Science and Technology and the Ministry of Health, Labour and Welfare Japan [20].

**Table 1:** Angiotensin II type I receptor blockers

**Generic name**	**Trade name**	**Number of cases of monotherapy**
Losartan	Nu-lotan®	214
Valsartan	Diovan®	266
Telmisartan	Micardis®	185
Candesartan	Blopress®	458
Olmesartan	Olmetec®	192

### Exposure and measurements

The baseline measurement period (non-exposure period) was defined as within 12 months before the start of ARB monotherapy. The exposure period (outcome measurement period) was defined as between 2 and 12 months after the start of ARB monotherapy. Laboratory data of the level of SUA for each subject were collected at the date nearest the start of ARB monotherapy in the baseline period, and at the date nearest 12 months after the start of ARB monotherapy in the exposure period. The mean exposure of losartan was 264.7 days, valsartan 245.3 days, telmisartan 235.9 days, candesartan 248.9 days, and olmesartan 234.5 days.

### Data elements

For each patient, we collected information of patient demographics (age and sex), medical history, use of medication, and laboratory results as baseline covariates for adjustment. Medical history included cerebrovascular disease (ICD-10 code; I60-69), ischemic heart disease (I20-I25), other heart disease (I30-I52), malignant neoplasm (C00-C97), thyroid gland disorder (E00-E07), rheumatoid disease (M5, M6), liver disease (K70-K77), kidney disease (N00-N19), hyperlipidemia (E78.0-E78.5), and proteinuria diagnosed in the 365 days preceding the first date of prescription of ARB. Drugs used during the 60 days before the start of ARB monotherapy included oral hypoglycemic drugs, immunosuppressive drugs, lipid-lowering drugs (including statins, fibrates, and other lipid-lowering drugs), thyroids drugs, antipsychotics, antithrombotic drugs, chemotherapeutic drugs, liver disease therapeutics, chronic kidney disease (CKD) therapeutics drugs, steroids, non-steroidal anti-inflammatory drugs (NSAIDs), proton pump inhibitors, and histamine H2 receptor blockers.

### Statistical analysis

All statistical analyses were performed with SAS software, version 9.2 (SAS Institute Inc., Cary, NC). This is a retrospective observational study. Because the non-randomized subjects had inherent issues of selection bias and confounding factors, we used a propensity-score method to minimize selection bias, and a multivariate regression model to measure the effect of ARBs on SUA while controlling for baseline confounders. To adjust for differences in baseline covariates among ARB users, we performed a propensity-score weighting technique [21-23]. This method is also known as the inverse probability of treatment weighted (IPTW) estimator, described by Robins and colleagues [24]. The details of the IPTW method are described elsewhere [22,24]. In brief, the propensity score was calculated using a logistic regression model that includes the predictor variable (ARBs) as an outcome and all baseline covariates (including sex, age, medical history, and previous drugs) as shown in Table 2. After the propensity score was constructed, we calculated the 'propensity score weight’ as the inverse of the propensity score. Then, propensity score-weighted (IPTW-adjusted) analysis was performed to balance the difference in baseline characteristics among ARBs and minimize the selection bias. We used IPTW-adjusted chi-squared test for categorical data, and an IPTW-adjusted linear regression model with Tukey’s post-hoc analysis for continuous variables to compare the difference in baseline characteristics among ARB users. We used multivariate regression models with Dunnett-Hsu post-hoc analysis to compare the mean values of SUA at baseline and during the exposure period in users of each ARB. We used an IPTW-adjusted multivariate regression model with Tukey’s post-hoc analysis to compare the mean change from the baseline value to the exposure value among ARB users. The model was adjusted for age, sex, medical history and previous medication, as listed in Table 2. All reported p-values are two sided. A result was considered statistically significant if the p value was less than 0.05.

**Table 2:** Baseline characteristics

**Characteristics**	**Number of the patients**					
	**Losartan**	**Valsartan**	**Telmisartan**	**Candesartan**	**Olmesartan**	
**Total number of patients**	214	266	185	458	192	
**Men**	113	172	113	272	127	
**Medical History**						
**Cerebrovascular disease**	61	86	63	135	47	
**Ischemic heart disease**	70	93	69	168	50	
**Other heart disease**	54	75	43	118	49	
**Malignant neoplasm**	104	136	87	252	105	
**Thyroid disease**	76	78	67	149	69	
**Rheumatoid disease**	56	33	35	68	19	
**Liver disease**	111	148	99	265	104	
**Kidney disease**	127	132	116	226	90	
**Hyperlipidemia**	128	167	112	287	113	
**Proteinuria**	96	98	71	170	73	
**Current Medication**						
**Oral hypoglycemic drugs**	46	78	62	129	69	
**Immnosupressive drugs**	16	6	6	11	5	
**Lipid-lowering drugs**	86	99	65	183	69	
**Statin**	69	75	53	134	52	
**Fibrate**	6	11	6	26	8	
**Other lipid-lowering drugs**	21	18	13	42	13	
**Thyroid drugs**	8	5	6	16	2	
**Antipsychotic drugs**	22	11	6	23	7	
**Antithrombotic drugs**	72	87	63	133	44	
**Chemotherapeutics drugs**	3	9	4	21	4	
**Liver disease therapeutics**	15	10	4	15	13	
**CKD therapeutic drugs**	6	0	0	2	0	
**Steroids**	40	23	24	49	13	
**NSAIDs**	82	96	57	132	52	
**Proton pump inhibitors**	47	34	35	73	35	
**H2 blockers**	48	38	26	75	23	

*NSAID*, Non-steroidal anti-inflammatory drug; *CKD*, Chronic kidney disease.

## Results

Tables 2 and 3 show the characteristics of the patients who had been treated with ARB monotherapy, before and after IPTW adjustment. Before adjustment, there were significant differences among users of different ARB in the mean values of age, frequency of men, prevalence of rheumatoid and kidney disease, and the frequency of use of oral hypoglycemic drugs, immunosuppressive drugs, antipsychotics, liver disease therapeutics, steroids, NSAIDs, proton pump inhibitors, and H2 blockers (Table 3). After adjustment, there was no statistically significant difference in all covariates among the ARB users. After adjustment, the mean age of losartan users was 63.4, valsartan 62.7, telmisartan 63.4, candesartan 62.4, and olmesartan 62.9 years. After adjustment, the frequency of men was 60.0% in losartan users, 61.2% in valsartan users, 59.3% in telmisartan users, 60.9% in candesartan users, and 55.8% in olmesartan users.

**Table 3:** Baseline characteristics before and after IPTW adjustment (percent distributions)

**Characteristics**		**†Percent distribution**											
		**Before adjustment**						**After IPTW adjustment**					
		**Losartan**	**Valsartan**	**Telmisartan**	**Candesartan**	**Olmesartan**	**p value**	**Losartan**	**Valsartan**	**Telmisartan**	**Candesartan**	**Olmesartan**	**p value**
**Age (years, mean ± SE)**		60.1 ± 0.8	63.4 ± 0.8	63.2 ± 0.9	63.2 ± 0.6	61.2 ± 0.9	0.0084*	63.4 ± 0.8	62.7 ± 0.8	63.4 ± 0.9	62.4 ± 0.6	62.9 ± 0.9	0.8485
**Men**		52.8	64.7	61.1	59.4	66.2	0.0394*	60.0	61.2	59.3	60.9	55.8	0.7809
**Medical History**													
	**Cerebrovascular disease**	28.5	32.3	34.1	29.5	24.5	0.2667	28.5	31.0	32.5	29.3	30.1	0.9066
	**Ischemic heart disease**	32.7	35.0	37.3	36.7	26.0	0.0917	30.1	34.0	35.4	34.7	30.8	0.6735
	**Other heart disease**	25.2	28.2	23.2	25.8	25.5	0.8313	26.7	26.3	25.1	25.7	23.3	0.9434
	**Malignant neoplasm**	48.6	51.1	47.0	55.0	54.7	0.2735	52.0	52.0	52.6	52.4	52.1	0.9999
	**Thyroid disease**	35.5	29.3	36.2	32.5	35.9	0.4354	31.6	32.0	34.0	33.8	35.0	0.9302
	**Rheumatoid disease**	26.2	12.4	18.9	14.9	9.9	<.0001*	17.5	14.5	15.8	16.4	16.0	0.9361
	**Liver disease**	51.9	55.6	53.5	57.9	54.2	0.6296	52.8	55.3	57.0	54.5	53.5	0.9303
	**Kidney disease**	59.4	49.6	62.7	49.3	46.9	0.002*	47.7	52.1	54.3	52.6	51.8	0.7232
	**Hyoerlipidemia**	59.8	62.8	60.5	62.7	58.9	0.8559	58.3	61.0	61.1	60.7	61.8	0.9594
	**Proteinuria**	44.9	36.8	38.4	37.1	38.0	0.3621	38.5	37.5	35.5	38.5	41.8	0.7941
**Current Medication**													
	**Oral hypoglycemic drugs**	21.5	29.3	33.5	28.2	35.9	0.0155*	31.0	30.7	28.8	28.6	29.9	0.9614
	**Immunosuppressive drugs**	7.5	2.3	3.2	2.4	2.6	0.0077*	3.7	2.6	3.7	3.1	2.7	0.9372
	**Lipid-lowering drugs**	40.2	37.2	35.1	40.0	35.9	0.6958	35.1	39.0	39.0	39.0	38.1	0.8877
	**Statin**	32.2	28.2	28.7	29.3	27.1	0.8211	27.3	30.2	29.6	29.5	28.6	0.9659
	**Fibrate**	2.8	4.1	3.2	5.7	4.2	0.4381	3.1	4.2	4.8	4.5	4.8	0.8962
	**Other lipid-lowering drugs**	9.8	6.8	7.0	9.2	6.8	0.5729	8.3	8.9	9.2	8.2	7.0	0.9402
	**Thyroid drugs**	3.7	1.9	3.2	3.5	1.0	0.3284	2.6	2.2	2.7	3.1	2.7	0.971
	**Antipsychotic drugs**	10.3	4.1	3.2	5.0	3.7	0.007*	5.7	3.8	4.1	4.6	7.2	0.5156
	**Antithrombotic drugs**	33.6	32.7	34.1	29.0	22.9	0.0783	27.7	30.7	30.6	30.0	29.6	0.9579
	**Chemotherapeutic drugs**	1.4	3.4	2.2	4.6	2.1	0.1554	3.1	3.1	4.9	3.3	3.1	0.8304
	**Liver disease therapeutics**	7.0	3.8	2.2	3.3	6.8	0.0405*	4.4	4.2	3.0	3.8	5.4	0.834
	**CKD therapeutic drugs**	2.8	0.0	0.0	0.4	0.0	0.0003*	0.6	0.0	0.0	0.9	0.0	0.236
	**Steroids**	18.7	8.7	13.0	10.7	6.8	0.0012*	9.7	10.1	11.4	10.6	10.7	0.9859
	**NSAIDs**	38.3	36.1	30.8	28.8	27.1	0.0347*	29.8	32.1	32.7	31.2	31.1	0.9771
	**Proton pump inhibitors**	22.0	12.8	18.9	15.9	18.2	0.087*	18.1	17.5	16.2	16.9	19.6	0.9208
	**H2 blockers**	22.4	14.3	14.1	16.4	12.0	0.0395*	14.9	15.6	19.1	15.9	18.4	0.7162

*IPTW*, Inverse probability of treatment weighted; *SE*, Standard error; *NSAID*, Non-steroidal anti-inflammatory drug; *CKD*: Chronic kidney disease. *: p < 0.05 (chi-squared test for categorical data, linear regression model for continuous data). †: Data are percent distribution of patients unless otherwise stated.

Table 4 shows the results of laboratory tests at baseline and during the exposure period. In losartan users, the mean level of SUA was significantly decreased in the exposure period compared with the baseline level. In users of other ARBs, valsartan, telmisartan candesartan, and olmesartan, the mean levels of SUA were significantly increased in the exposure period compared with those in the baseline.

**Table 4:** Adjusted mean level of SUA at baseline and during exposure period

**ARB**	**Baseline**		**Exposure**		**p value**
	**Mean**	**95% CI**	**Mean**	**95% CI**	
Losartan	5.18	(5.03, 5.32)	5.04	(4.90, 5.19)	0.0194*
Valsartan	5.30	(5.15, 5.45)	5.49	(5.34, 5.63)	0.0012*
Telmisartan	5.34	(5.18, 5.49)	5.47	(5.32, 5.63)	0.0253*
Candesartan	5.54	(5.43, 5.65)	5.68	(5.57, 5.79)	0.0011*
Olmesartan	5.39	(5.22, 5.56)	5.58	(5.41, 5.75)	0.0013*

*ARB*, Angiotensin II type I receptor blocker; *CI*, Confidence interval. *: p < 0.05 (exposure period vs baseline, Dunnett-Hsu post-hoc analysis). Analyses were adjusted for covariates including age, sex, medical history and previous medication.

Table 5 shows the mean change in SUA level during the exposure period from baseline, after IPTW adjustment. The reduction of SUA level in losartan user was significantly greater in comparison with that in valsartan, telmisartan, candesartan, and olmesartan users. There was no significant difference in the increase of SUA level among the users of ARBs other than losartan; valsartan, telmisartan, candesartan, and olmesartan.

**Table 5:** Adjusted mean change of SUA level from baseline to exposure period

**ARB**	**Mean**	**95% CI**	**p value**			
			**vs Losartan**	**vs Valsartan**	**vs Telmisartan**	**vs Candesartan**
ΔLosartan	-0.124	(-0.243, -0.0055)	-	-	-	-
ΔValsartan	0.186	(0.0824, 0.289)	0.0012*	-	-	-
ΔTelmisartan	0.150	(0.0239, 0.275)	0.0172*	0.9926	-	-
ΔCandesartan	0.153	(0.0755, 0.231)	0.0013*	0.9884	1	-
ΔOlmesartan	0.166	(0.0413, 0.290)	0.0089*	0.9992	0.9998	0.9998

*ARB*, Angiotensin II type I receptor blocker; *CI*, Confidence interval. Δ: mean change in SUA level during exposure period from baseline. *: p < 0.05 (p value among ARBs, Tukey’s post-hoc analysis). Analyses were adjusted for covariates including age, sex, medical history and previous medication.

## Discussion

In this study, we evaluated and compared the effect of long-term monotherapy, up to one year, among five ARBs on SUA in hypertensive patients with type 2 DM. The mean level of SUA after treatment with losartan significantly decreased compared with the baseline. The mean level of SUA after treatment with other ARBs (valsartan, telmisartan, candesartan, and olmesartan) significantly increased compared with baseline. The reduction of SUA level from baseline in losartan users was significantly greater than that in other ARB users. This study suggests that, among the five ARBs, losartan had the most beneficial effect on SUA in hypertensive patients with type 2 diabetes mellitus.

It is known that losartan decreases the level of SUA in clinical practice and in animals. In clinical practice, some studies have reported a lowering effect of losartan on SUA level. It was reported that losartan significantly lowered SUA compared to placebo in patients with type 2 diabetes and nephropathy [9]. In patients with hypertension, 12-week treatment with losartan decreased the mean level of SUA compared with baseline [14]. In patients with mild to moderate hypertension, the mean level of SUA was significantly decreased from baseline after 12 weeks of treatment with losartan [25]. A study using xenopus oocytes, an in vitro study, and administration in hypertensive patients have revealed that losartan decreases SUA level via inhibition of URAT 1, which is the transporter of uric acid re-absorption in the proximal renal tubule [11-13]. Supporting these previous reports, our study indicated that long-term monotherapy with losartan has a beneficial effect on SUA level in mildly to moderately hypertensive patients with type 2 DM. Because hypertension, DM and hyperuricemia often coexist, therapy with losartan is suitable for hypertensive patients with type 2 DM.

In a comparison of ARBs, it has been shown that the reduction of SUA level by losartan is stronger than that by other ARBs. In patients with hypertension and serum uric acid ≥ 7 mg/dL, the mean level of SUA was significantly decreased after 24 weeks of losartan treatment compared with candesartan treatment [15]. In one study, losartan but not irbesartan significantly lowered SUA level compared to placebo in patients with type 2 diabetes and nephropathy [8]. The risk of onset of gout, which is strongly related to SUV level, has been reported to be lower in losartan users than in other ARB or CCB users [25,26]. In this multiple comparison study, we showed that losartan had the most beneficial effect on SUA level among five ARBs in hypertensive patients with type 2 DM. Based on these clinical findings, losartan should be preferentially used in patients with hypertension, especially in those with comorbid disease of hyperuricemia or gout, over other ARBs.

Some in vitro studies have investigated the different effects of ARBs on URAT1, which may explain the variable effects of ARBs on SUA level. Candesartan, olmesartan and valsartan did not show a cis-inhibitory effect but showed a trans-stimulatory effect on URAT1, potentially leading to an increase of SUA level [12]. Corresponding with these in vitro studies, several clinical studies have reported that some ARBs increased SUA level. In patients with hypertension, 12-week treatment with valsartan increased the mean level of SUA compared with baseline [14]. In patients with coronary artery disease, the mean SUA level in valsartan users was increased compared with the baseline [27]. In patients with mild to moderate hypertension, the mean level of SUA was significantly increased after 12 weeks of treatment with candesartan [25]. In this study, the mean SUA level in candesartan, olmesartan and valsartan users was increased in comparison with baseline, and there was no significant difference in the mean change of SUA level in the exposure period from baseline among these ARBs. On the other hand, in the study using xenopus oocytes, losartan and telmisartan exhibited a cis-inhibitory effect on uric acid transport via URTA1, which means a reduction of reabsorption of uric acid [12]. To our knowledge, however, there is no clinical report that telmisartan may decrease SUA level, whereas some clinical studies have shown a lowering effect of losartan on SUA level. In patients with hypertension, high-dose treatment with telmisartan for three months significantly increased SUA level [28]. In this study, we showed that long-term monotherapy with telmisartan increased SUA level. The reason for this discrepancy between in vitro and clinical study outcomes is unclear. The contribution of other mechanisms, e.g., disturbance of urinary excretion, which may be predominant over the inhibitory effect on URTA1 in telmisartan users, cannot be excluded. Concerning ARBs other than losartan, our findings suggest that regular checks of SUA level are recommended in patients treated with candesartan, olmesartan, valsartan or telmisartan.

Our study has several limitations. First, the retrospective and non-randomized nature of the design entailed inherent issues of selection bias and confounding. We used rigorous statistical methods to control potential confounding variables between ARB users, including IPTW adjustment and a multivariate regression model. However, their ability to control for differences was limited to variables that were available or measurable. An important potential confounding factor that could affect the results of this study is renal function status. This analysis was adjusted for covariates including kidney disease, proteinuria and medication for CKD. However, other biochemical tests such as serum creatinine and urea nitrogen were not estimated in this analysis because there were some missing data in the study population. Other potential confounding factors that could not be considered in this database study are alcohol intake, smoking, body mass index (BMI) and muscle mass index (MMI). Furthermore, the possibility that the findings of comparison of the baseline and exposure period in each treatment group may be confounded by other variables should be considered when interpreting the results. Therefore, the findings of our study, based on a non-randomized design, call for further studies, such as similar analyses of larger databases, prospective population-based studies, and randomized clinical trials, for confirmation. Second, we did not fix the daily dosage of ARBs, because the achievement of BP goal requires various doses of an agent across different individuals or even in the same individual in clinical practice. This study was not designed to assess the effects of ARBs at each dosage, because it is difficult to determine whether or not pharmacodynamics are dose-dependent in clinical settings. However, we consider that the findings of our study, using a sophisticated statistical method in a real-world setting, are reliable and will be informative for clinicians.

## Conclusion

The results of the present study suggested that losartan had the most beneficial effect on SUA level among five ARBs; losartan, valsartan, candesartan, telmisartan, and olmesartan, at least up to one year. Our study provides evidence of the long-term effect of various ARBs on SUA level in hypertensive patients with type 2 DM.

## Abbreviations

ARB: Angiotensin type II receptor blocker; CCB: Calcium channel blockers; CDW: Clinical data warehouse; CKD: Chronic kidney disease; DM: Diabetes mellitus; IPTW: Inverse probability treatment weighting; NSAID: Non-steroidal anti-inflammatory drug; NUSM: Nihon University School of Medicine; SUA: Serum uric acid; URAT1: Urate transporter1.

## Competing interests

The authors declare that they have no competing interests.

## Authors’ contributions

YN and YT conceived the study and participated in its design. YN performed the statistical analyses. YN and YT drafted the manuscript. TN, NS, NK and SA interpreted the data. All authors have read and approved the final manuscript.
